# EORTC QLU-C10D value sets for Austria, Italy, and Poland

**DOI:** 10.1007/s11136-020-02536-z

**Published:** 2020-05-26

**Authors:** E. M. Gamper, M. T. King, R. Norman, F. Efficace, F. Cottone, B. Holzner, G. Kemmler, the European Organisation for Research and Treatment of Cancer (EORTC) Quality of Life Group

**Affiliations:** 1 Department of Psychiatry, Psychotherapy and Psychosomatics, University Hospital Psychiatry II, Medical University of Innsbruck, Innsbruck, Austria; 2 School of Psychology, Psycho-Oncology Co-operative Sydney, University of Sydney, Camperdown, NSW 2006 Australia; 3 School of Public Health, Curtin University, Perth, Australia; 4 Italian Group for Adult Hematologic Diseases (GIMEMA), Data Center & Health Outcomes Research Unit, Rome, Italy; 5 Department of Psychiatry, Psychotherapy and Psychosomatics, University Hospital Psychiatry I, Medical University of Innsbruck, Innsbruck, Austria

**Keywords:** EORTC QLU-C10D, Cancer-specific, Health utilities, Austria, Poland, Italy, EORTC QLQ-C30, Discrete choice experiment

## Abstract

**Objective:**

To develop Austrian, Italian, and Polish general population value sets for the EORTC QLU-C10D, a cancer-specific utility instrument based on the EORTC QLQ-C30, and to descriptively compare their index scores for distinct health states.

**Methods:**

The QLU-C10D descriptive system comprises 10 health attributes and each can take on 4 levels. A standardised and pre-tested methodology has been applied for valuations including a web-based discrete choice experiment (DCE). It was administered in 1000 general population respondents per country recruited via online panels, aiming at representativeness for core socio-demographic variables.

**Results:**

In all three countries, the attributes with the largest impact on utility were physical functioning, pain, and role functioning. Cancer-specific dimensions with the largest impact were nausea and fatigue or bowel problems. Utility values of the worst health state (i.e. severe problems on all 10 dimension) were -0.111 (Austria), 0.025 (Italy), and 0.048 (Poland). Country-specific utilities differed for a selection of health states across the continuum. Austrian utilities were systematically lower for moderately and severely impaired health states.

**Conclusion:**

QLU-C10D cancer-specific utilities can now be calculated in three more countries. Differences between countries indicate that careful consideration is required when using non-country-specific value sets in economic evaluations.

**Electronic supplementary material:**

The online version of this article (10.1007/s11136-020-02536-z) contains supplementary material, which is available to authorized users.

## Introduction

Cost-utility analyses (CUAs) are an essential source of information for rational decision-making in resource allocation in health care. Their primary outcome is quality adjusted life years (QALYs), a parameter which integrates survival time and the “value” of a specific health state: the health utility. Health utilities are cardinal values that represent an individual’s preferences for specific health states, with “0” considered equivalent to death and “1” reflecting perfect health. Among the different preference-based methods used to obtain utilities, multi-attribute utility instruments (MAUIs) are popular. Well-known and frequently used MAUIs include the EQ-5D [1, 2] and the SF-6D [3]. Like most MAUIs, these are generic, i.e. they cover very general health aspects (such as mobility, pain, or self-care) which makes them applicable to a broad range of different health conditions. This breadth is valuable, but may come at the price of missing specific health issues relevant to certain conditions, such as nausea and vomiting in cancer [4, 5]. However, utility instruments using a disease-specific health state description system are relatively scarce.

Non-preference-based disease-specific quality of life (QOL) measures are widely used in clinical research. By definition, they include aspects of health relevant to a certain disease, which arguably may be more sensitive to clinical changes [5]. However, they do not allow utility scoring, and therefore cannot be used in cost-utility analysis.

The recent development of the EORTC Quality of Life Utility-Core 10 Dimensions (QLU-C10D) [6] contributes to closing this gap, as it is a MAUI based on the EORTC Quality of Life Questionnaire Core 30 (QLQ-C30) [7], the most widely used QOL profile measure in clinical oncology research [8]. It helps to overcome the lack of disease-specific utility instruments in the field of oncology by providing a health state classification system and utility algorithm using 13 key items of the parent instrument, covering 10 QOL dimensions.

QLU-C10D valuations are currently being performed for a range of countries and tariffs have already been published for Australia [9], Canada [10], Germany [11], and the UK [12]. In the present study, we aim to determine the utility weights for health states of the Austrian, Italian, and Polish version of the QLU-C10D. Furthermore, we descriptively compare the utilities of the three countries for selected QLU-C10D health states.

## Methods

For QLU-C10 valuations a standardised survey has been developed consisting of a discrete choice experiment (DCE) for the valuation of health states as its core element, and of feedback questions on the participants’ experience of the DCE, self-report instruments on QOL (EORTC QLQ-C30, EQ-5D) and distress (Kessler K-10), and questions on basic socio-demographic and basic clinical information [13]. Before adopting the approach as method of choice for QLU-C10D valuations, feasibility and reliability have been established using quantitative and qualitative methods. The wording of the DCE tasks and different layouts have been pre-tested in general population respondents which showed that although perceived difficult the tasks were considered manageable [14]. Utility weights resulting from the DCE showed to be unbiased by the ordering of attributes in the DCE [15] and stable within respondents over time [16].

The QLU-C10D descriptive system [6] consist of 13 of the 30 items of the parent instrument EORTC QLQ-C30 [17] covering the 10 QOL domains physical functioning, role functioning, social functioning, emotional functioning, pain, fatigue, sleep disturbances, appetite loss, nausea, and bowel problems. Each can take on 4 levels from the best level “not at all” (coded 1) to the worst level “very much” (coded 4) (see Table 1 for entire health state classification system). For example a health state with “very much” problems in physical and role functioning and “no problems” on other QLU-C10D domains would be coded 4411111111. The combination of domain and level therefore is able to describe a total of 4^10^ = 1,048,576 unique health states. These are the health states for which preferences need to be obtained in the valuation using the DCE.

**Table 1:** Health state classification system of the QLU-C10D

Dimension	Level	Stem	Descriptor	EORTC QLQ-C30 item levels
Physical functioning	1	You have	No trouble taking a long walk outside of the house	Item 2 (long walk) = 1
	2		No trouble taking a short walk outside of the house, but at least a little trouble taking a long walk	Item 3 (short walk) = 1 AND Item 2 ≥ 2
	3		At least a little trouble taking a short walk outside of the house, and at least a little trouble taking a long walk	Item 3 = 2 AND Item 2 ≥ 2
	4		Quite a bit or very much trouble taking a short walk outside the house	Item 3 ≥ 3 AND Item 2 ≥ 2
Role functioning	1	You are limited in pursuing your work or other daily activities…	Not at all	Item 6 = 1
	2		A little	Item 6 = 2
	3		Quite a bit	Item 6 = 3
	4		Very much	Item 6 = 4
Social functioning	1	Your physical condition or medical treatment interferes with your social or family life	Not at all	Items 26 AND 27 = 1
	2		A little	Items 26 OR 27 = 2
	3		Quite a bit	Items 26 OR 27 = 3
	4		Very much	Items 26 OR 27 = 4
Emotional functioning	1	You feel depressed	Not at all	Item 24 = 1
	2		A little	Item 24 = 2
	3		Quite a bit	Item 24 = 3
	4		Very much	Item 24 = 4
Pain	1	You have pain	Not at all	Item 9 = 1
	2		A little	Item 9 = 2
	3		Quite a bit	Item 9 = 3
	4		Very much	Item 9 = 4
Fatigue	1	You feel tired	Not at all	Item 18 = 1
	2		A little	Item 18 = 2
	3		Quite a bit	Item 18 = 3
	4		Very much	Item 18 = 4
Sleep	1	You have trouble sleeping	Not at all	Item 11 = 1
	2		A little	Item 11 = 2
	3		Quite a bit	Item 11 = 3
	4		Very much	Item 11 = 4
Appetite	1	You lack appetite	Not at all	Item 13 = 1
	2		A little	Item 13 = 2
	3		Quite a bit	Item 13 = 3
	4		Very much	Item 13 = 4
Nausea	1	You feel nauseated…	Not at all	Item 14 = 1
	2		A little	Item 14 = 2
	3		Quite a bit	Item 14 = 3
	4		Very much	Item 14 = 4
Bowel Problems^a^	1	You…	do not have constipation or diarrhoea at all	Items 16 AND 17 = 1
	2		have a little constipation or diarrhoea	Items 16 OR 17 = 2
	3		have constipation or diarrhoea quite a bit	Items 16 OR 17 = 3
	4		have constipation or diarrhoea very much	Items 16 OR 17 = 4
Duration	1	You will live in this health state for…	1 year, and then die	Not applicable
	2		2 years, and then die	Not applicable
	3		5 years, and then die	Not applicable
	4		10 years, and then die	Not applicable

^a^Derived from two separate dimensions of the QLQ-C30 (diarrhoea and constipation)

**Fig. 1:**
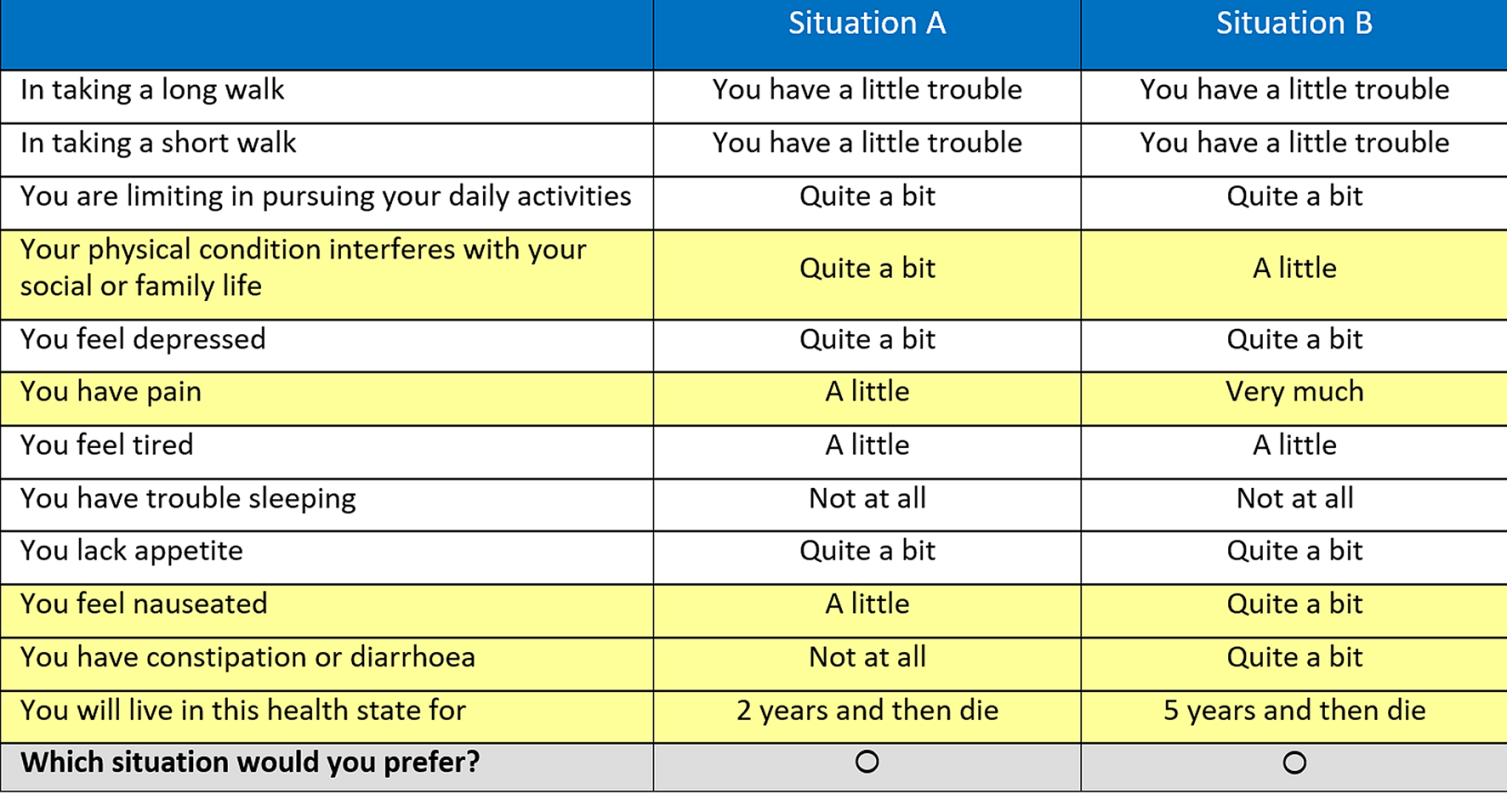
English example choice set for discrete choice experiment valuation task

The DCE asks respondents to choose between pairs of (hypothetical) health states which are described by 11 attributes: the 10 domains of the QLU-C10D (in a randomised order across participants to control for any potential dimension ordering effect) and a survival time of 1, 2, 5, or 10 years. These durations were selected to be plausible to most respondents, with enough spread to ensure discrimination between them. Each respondent is presented with 16 binary choice sets randomly selected from a choice of 960 sets. Only five attributes differ between the health states in each choice set to minimise the cognitive burden. An example choice set as presented to respondents is shown in Fig. 1 for details on the DCE design please refer to King et al. (2018) [9].

As with previous EORTC QLU-C10D valuations in other countries, recruitment and survey administration were performed web-based by survey engine (www.surveyengine.com), a survey company specialising in choice experiments. The survey was sent out as a weblink for the respondents to complete at their leisure. The approached potential respondents were members of an online panel of persons willing to complete surveys for a small payment. Survey Engine and its panel providers comply with the International Code on Market, Opinion and Social Research and Data Analytics (www.esomar.org).

In each country, we aimed to recruit 1000 respondents from the general population, aged between 18 and 80. Quota sampling by age and sex was applied to ensure that these variables were representative. The representativeness of educational level, marital status, and chronic disease (yes/no) were checked a posteriori by comparison with national census data. Data collection for all countries were performed in the years 2016–2017.

For the present study, validated translations of all required questionnaires were available. Also the entire Austrian survey was already available in German (see [11]). With regard to the QLU-C10D health state description system attribute descriptions were already available in all languages as they were taken from the parent instrument QLQ-C30. The respective questions are in past tense and were changed into a statement in the present tense (“Did you feel tired?” to “You feel tired.”). This and the translation of the remaining survey text (section headings and brief section summaries, DCE task descriptions, thank you notes) was contracted to translators, forward and backward translations were performed including in-country persons and pilot testing in convenience samples of 3–5 persons were conducted.

It has to be noted that for Austria, a revised German response format of the QLQ-C30 was used which has also been used for QLU-C10D valuations in Germany [11]. The reason for revision was that the original German wording for the category “quite a bit” (“mäßig”) is suspected to express a lower severity than the English version [18] and a revised German wording for this response level (“ziemlich”) is currently being investigated within the EORTC QLG [19]. The new translation appears to be a closer approximation of the severity level expressed by “quite a bit” (personal communication with study PI) but results are not yet published. The health states for QLU-C10D valuations for Austria are already based on the adapted German version, and consequently the utility weights presented here for Austria are valid for this version, which is abbreviated as “QLU-C10D Austria V2”.

Utilities score across countries were descriptively compared across a selection of QLU-C10D health states covering a continuum between best (i.e. 1111111111) and worst (i.e. 4444444444).

### Statistical methods

#### Representativeness and feedback

Comparisons of socio-demographic and clinical characteristics with national census data [20–25] were performed by Chi-square tests. Feedback questions were analysed by descriptive statistical methods.

### Utility estimation

Country-specific utility weights for the QLU-C10D were determined by conditional logistic regression using the method proposed by Bansback et al. (2012) [26]. The basic model for the utility of option j (scenario A or B) in choice set s for respondent i is given by

$$U_{isj} = \alpha TIME_{isj} + \beta X^{\prime}_{isj} TIME_{isj} + \varepsilon_{isj}$$

where TIME_isj_ is the survival time presented in option j and X’_isj_ is a set of dummy variables related to the levels of QOL dimensions in the corresponding health state. The errors ε_isj_ were assumed to be independent and identically Gumbel distributed. The parameters α (scalar) and β (vector) were estimated by conditional logistic regression. To allow for within-subject correlations across different choice sets, a random subject-level term was included in the model using generalised estimation equation (GEE) models with first-order autoregressive covariance structure and a logit link function; this procedure gave rise to almost identical results as the conditional logistic regression analysis approach with a clustered sandwich estimator used in a previous study, implemented using STATA for the Australian QLU-C10D valuation data [9]. For QOL domains in which coefficients for levels did not show a monotonically increasing pattern with increasing severity levels, non-monotonic levels were combined; this is a common approach [3, 9]. All adjustments were conducted at once based on the raw coefficients; we checked the new results for potential non-monotonicities, but none were left. GEE model coefficients were then converted into utility decrements consisting of the ratio of the health state parameters b and the time coefficient a to reflect the trade-off between health-related QOL and length of life [26].

Statistical analyses were run using SPSS v24 and Stata v13.

### Power considerations

Sample size determination was based on the confidence interval (CI) for the estimated utility decrements. Building on the findings of King et al. (2018) [9] and allowing for the possibility of a slightly larger spread due to a more heterogeneous response pattern (factor 1.2), the half-length d of the 95% CIs for the utility decrements for samples of size *N* = 1,000 was estimated to be < 0.05 ([u – d, u + d] with d ≤ 0.05. This sample size is towards the higher end of the spectrum of sample sizes used so far in DCEs [27].

## Results

### Sample characteristics

#### Complete cases and dropouts

**Fig. 2:**
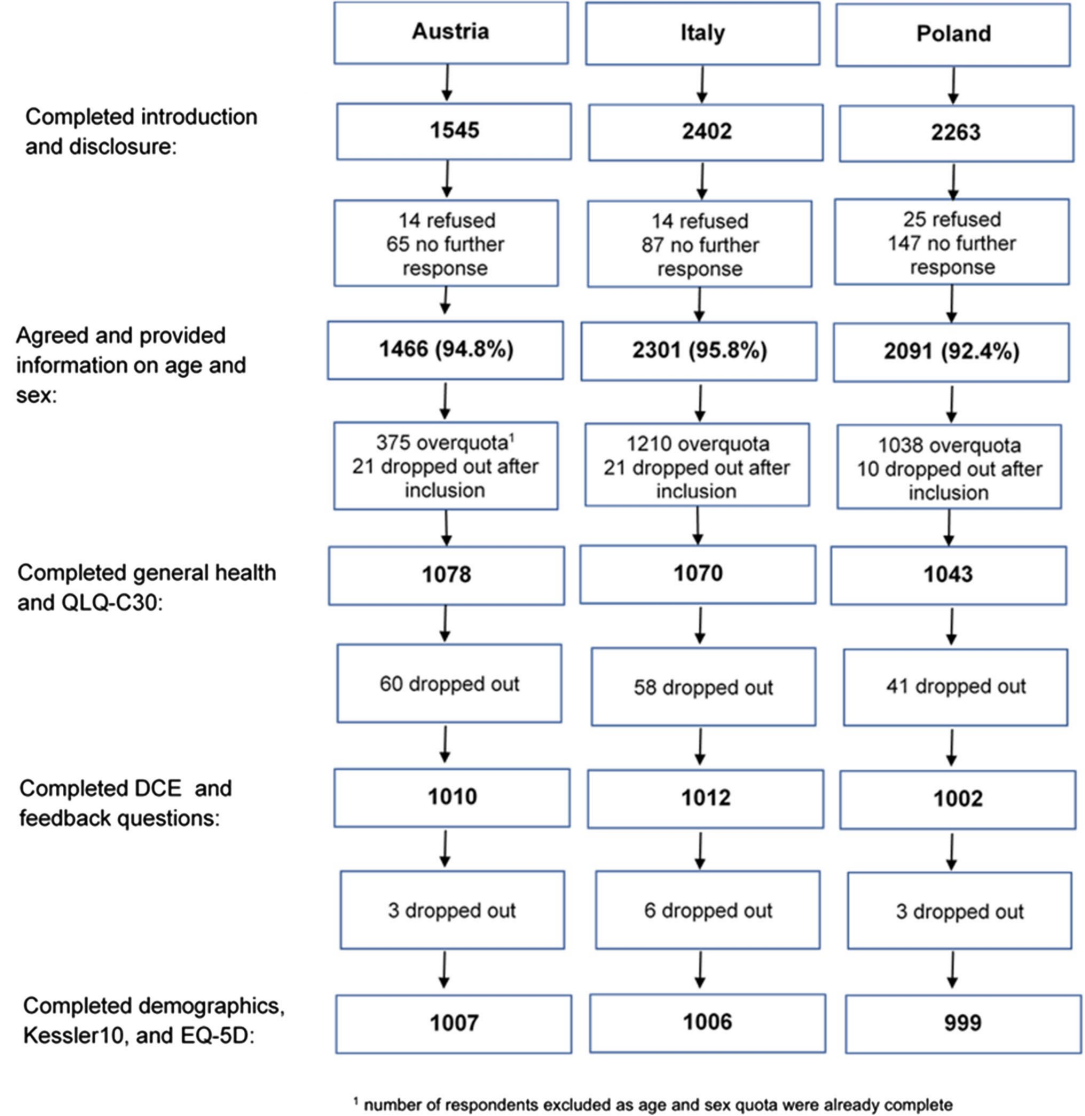
Flow diagram of number of respondents and dropouts per completed survey section and country

An N of 1000 was reached in each country within a period of 2 months. Figure 2 shows the flow diagram of respondents who entered the survey and the number of dropouts in each section.

#### Socio-demographic and clinical variables, representativeness, and feedback

An overview of the distribution of socio-demographic and clinical variables in the valuation samples is given in Table 2. The proportion of respondents with a high educational level was significantly larger in the valuation sample compared to census data (*p* < 0.01, χ^2^ > 6.7). In all countries the majority of the respondents regarded the presentation of the DCE as clear/very clear (70% Austria, 73% Italy, 58% Poland), indifferent ratings were more frequent in polish respondents (29% vs 17% in Austria and 18% in Italy) and the percentages of those considering it unclear were very similar between 9 and 13%. Up to half of the respondents with (50% Austria, 36% Italy, 36% Poland) considered it difficult to choose between the health states and 26%—31% found it to be easy. Details of the feedback are provided in Figure A1 (Online Resource 1).

**Table 2:** Distribution of socio-demographic and clinical characteristics and comparison with national statistics

Variable	Category	Italy (N = 1005)		Poland (N = 999)		Austria (N = 1007)	
		n	Percent	n	Percent	n	Percent
Age	18–30	159	15.8	198	19.7	210	21.1
	31–40	159	15.8	174	17.3	209	20.9
	41–50	205	20.4	193	19.2	165	16.5
	51–60	193	19.2	194	19.3	179	17.9
	61–70	155	15.4	140	13.9	151	15.1
	71–80	134	13.4	108	10.7	85	8.5
Sex	Male	492	49.0	492	48.9	483	48.3
	Female	513	51.0	515	51.1	516	51.7
Education	Compulsory	112	11.1↓	71	7.1↓	76	7.6↓
	Lower secondary	92	9.2↓	421	41.8↓	255	25.5
	Higher secondary (A-levels)	429	42.7↑	319	31.7↑	187	18.7
	Tertiary (university, polytechnic)	372	37.0↑	196	19.5↑	481	48.0↑
Marital status	Single	239	23.8	251	24.9	183	18.3
	Married/partner	656	65.3	594	59.0	690	68.9
	Divorced/separated	68	6.7	127	12.6	72	7.2
	Widowed	42	4.2	35	3.5	54	5.4
Chronic diseases	Yes	391	38.9↑	359	35.7	391	39.1
	No	614	61.1	648	64.3	608	60.9

↑More than 5 percentage points higher than in the general population

↓More than 5 percentage points lower than in the general population

#### Utility estimates for QLU-C10D Austria V2, Italy, and Poland

Decrements were largely monotonic within each QOL dimension, i.e. a higher impairment level was associated with a higher utility decrement. Any movement away from the response category “not at all” was associated with negative utility except for social functioning in Austria and Poland and emotional functioning in Austria and Italy. Non-monotonicity was observed in three domains: lack of appetite (all countries), fatigue (Poland, Italy), and sleep disturbances (Italy) but none was statistically significant. For final utility scoring the values have been monotonicity-adjusted and are provided in Table 3 for all countries. Table A2 (Online Resource 2) provides the unadjusted model raw scores.

**Table 3:** QLU-C10D utility weights (decrements)

Dimension	Level	Utility decrement^a^ (SE)		
		Austria^b^	Italy	Poland
Physical functioning	1 (not at all)	0	0	0
	2 (a little)	− 0.117 (0.021)	− 0.048 (0.027)	− 0.064 (0.025)
	3 (quite a bit)	− 0.234 (0.021)	− 0.204 (0.023)	− 0.149 (0.026)
	4 (very much)	− 0.316 (0.020)	− 0.299 (0.022)	− 0.272 (0.024)
Role functioning	1 (not at all)	0	0	0
	2 (a little)	−0.012 (0.017)	−0.021 (0.018)	−0.070 (0.020)
	3 (quite a bit)	−0.075 (0.016)	−0.075 (0.018)	−0.139 (0.023)
	4 (very much)	−0.138 (0.015)	−0.119 (0.017)	−0.196 (0.021)
Social functioning	1 (not at all)	0	0	0
	2 (a little)	0	−0.004 (0.018)	0
	3 (quite a bit)	−0.072 (0.013)	−0.041 (0.018)	−0.008 (0.019)
	4 (very much)	−0.103 (0.013)	−0.043 (0.016)	−0.033 (0.016)
Emotional functioning	1 (not at all)	0	0	0
	2 (a little)	0	0	−0.004 (0.020)
	3 (quite a bit)	0	−0.070 (0.016)	−0.020 (0.021)
	4 (very much)	−0.038 (0.011)	−0.117 (0.014)	−0.034 (0.018)
Pain	1 (not at all)	0	0	0
	2 (a little)	− 0.036 (0.016)	− 0.012 (0.018)	− 0.015 (0.020)
	3 (quite a bit)	− 0.112 (0.016)	− 0.074 (0.018)	− 0.067 (0.020)
	4 (very much)	− 0.182 (0.015)	− 0.125 (0.017)	− 0.125 (0.018)
Fatigue	1 (not at all)	0	0	0
	2 (a little)	− 0.028 (0.014)	− 0.013 (0.017)	− 0.012 (0.019)
	3 (quite a bit)	− 0.048 (0.015)	− 0.060 (0.019)	− 0.041 (0.016)
	4 (very much)	− 0.057 (0.014)	− 0.062 (0.016)	− 0.041 (0.016)
Sleep disturbances	1 (not at all)	0	0	0
	2 (a little)	−0.022 (0.014)	−0.027 (0.016)	−0.021 (0.019)
	3 (quite a bit)	−0.034 (0.016)	−0.046 (0.015)	−0.025 (0.020)
	4 (very much)	−0.039 (0.014)	−0.046 (0.015)	−0.038 (0.018)
Appetite loss	1 (not at all)	0	0	0
	2 (a little)	−0.049 (0.012)	−0.023 (0.016)	−0.016 (0.019)
	3 (quite a bit)	−0.049 (0.012)	−0.023 (0.015)	−0.049 (0.020)
	4 (very much)	−0.061 (0.013)	−0.023 (0.015)	−0.053 (0.019)
Nausea	1 (not at all)	0	0	0
	2 (a little)	−0.029 (0.014)	−0.037 (0.016)	−0.037 (0.018)
	3 (quite a bit)	−0.074 (0.015)	−0.080 (0.017)	−0.056 (0.020)
	4 (very much)	− 0.108 (0.014)	− 0.089 (0.016)	− 0.084 (0.017)
Bowel problems	1 (not at all)	0	0	0
	2 (a little)	− 0.022 (0.014)	− 0.025 (0.017)	− 0.034 (0.019)
	3 (quite a bit)	− 0.061 (0.015)	− 0.028 (0.017)	− 0.067 (0.020)
	4 (very much)	− 0.069 (0.014)	− 0.052 (0.016)	− 0.076 (0.018)

^a^Monotonicity-adjusted utility decrements

^b^Valid for QLU-C10D Austria V2 with revised response format “ziemlich” instead of “mäßig”

The largest decrements in Austria were found for physical functioning, followed by pain and role functioning. Among the cancer-specific symptoms, nausea received the highest decrement followed by bowel problems. The worst possible health state is − 0.111.

In Italy, the largest decrements were likewise found for physical functioning, then pain and role functioning, very closely followed by emotional functioning. The largest cancer-specific decrements were found for nausea and fatigue. The worst possible health state is 0.025.

In Poland, the highest decrements were again found for physical functioning, then role functioning, followed by pain; and the highest cancer-specific decrements were nausea and bowel problems. The worst possible health state is 0.048.

**Fig. 3:**
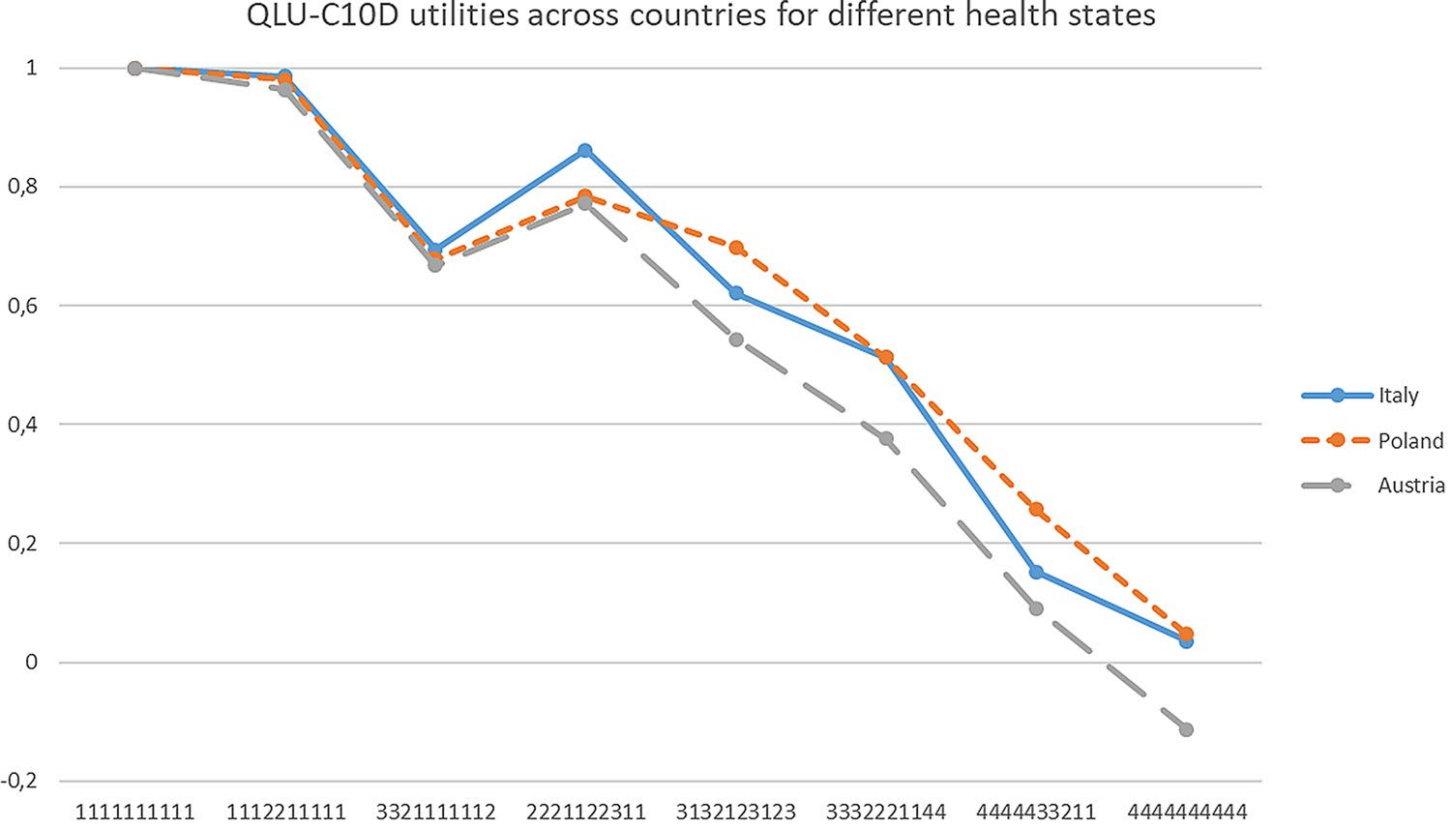
QLU-C10D utilities across countries for different health states

To get an overall impression of differences between countries on the utility level we compared index scores across a selection of unique QLU-C10D health states including best (i.e. 1111111111) and worst (i.e. 4444444444) health, some states with some mild and moderate impairments (1112211111, 3321111112, 2221122311, 3132123123) and some severe impairment (3332221144, 4444433211). It can be seen in Fig. 3 that utilities vary between countries for moderately and highly impaired health states and that for this spectrum of continuum Austrian utilities seem systematically lower.

#### QLU-C10D utility calculation for Austria, Italy, and Poland

**Fig. 4:**
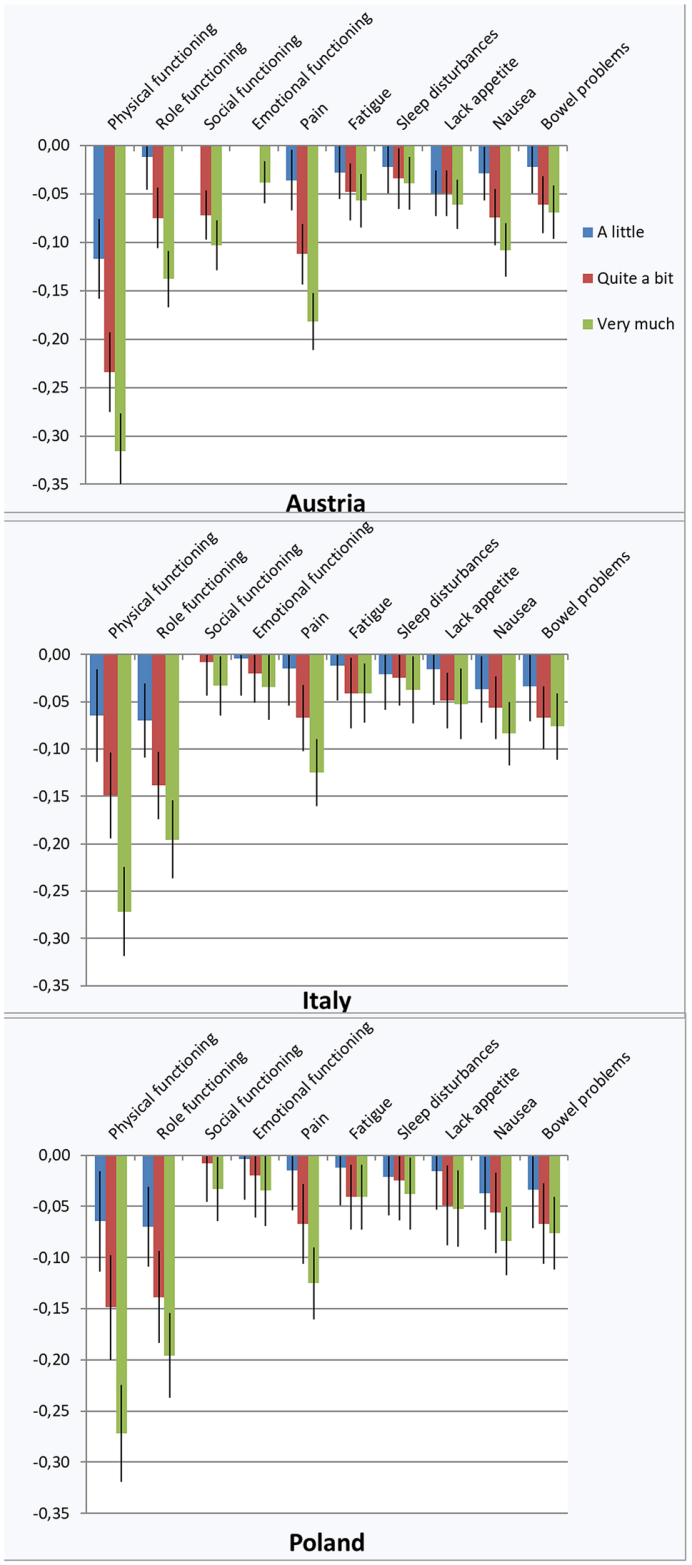
Utility decrements for the QLU-C10D versions Austria V2 (response category “ziemlich” instead of “mäßig”), Italy, and Poland

For the calculation of QLU-C10D utility scores responses to the respective QLQ-C30 responses are converted into QLU-C10D levels (see Table 1) and attached with the monotonicity-adjusted decrements presented in Table 3 and Fig. 4. The decrements for each level on each domain are subtracted from 1 to obtain the final utility score. For instance, a health state with very much problems with Role Functioning (level 4), moderate problems with Social Functioning (level 3), a little Fatigue (level 2), and no problems on other dimensions (level 1) would be coded 1431112111 and result in a utility score of *1-(0.138* + *0.072* + *0.028)* = *0.762* in Austria, a utility score of *1-(0.119* + *0.041* + *0.013)* = *0.853* in Italy, and a utility score of *1-(0.196* + *0.008* + *0.012)* = *0.784* in Poland.

Tables A3-A5 (Online Resources 3–5) provides an SPSS syntax code to implement the scoring.

## Discussion

The major advantage of the EORTC QLU-C10D is that it is based on the EORTC QLQ-C30, and therefore, cancer-specific utilities can be determined using data previously or prospectively collected with the QLQ-C30 in addition to its traditional QOL profile scoring.

The current study provides value sets for Austria, Italy, and Poland. In all three countries, respective guidelines consider QALYs to be one of the preferred outcome measures in economic evaluations [28]. Normally, generic instruments are used to obtain utilities for QALY calculation as a result of conceptual considerations; however, disease-specific measures can be more sensitive to clinical differences in the respective diseases [5]. Whether this assumption holds true for the QLU-C10D requires clinical validity evaluation. An indication for the potential relevance of cancer-specific symptoms are the decrements found for nausea, bowel problems and fatigue which are consistent with the Australian QLU-C10D valuation [9] as well as those in Germany, the UK [12], and Canada [10].

In overall, we found that the impact of some QOL dimensions on QLU-C10D utilities appeared to differ across countries. This was especially true for emotional functioning, for which decrements were clearly higher in Italy than in Austria and Poland. Austrian utilities tended to be systematically lower in moderately and severely impaired health states.

Possible explanations of observed differences between countries may include different health care systems or culture-specific attitudes towards health, which could impact the willingness to trade-off life time for QOL. A possible methodological cause may arise from slight differences in meaning through translation into different languages. Although the EORTC follows a rigorous translation procedures, there is some tangential evidence for differential item function (DIF) analyses conducted by Scott et al. [18, 29, 30], who found that some QLQ-C30 items functioned slightly differently in a range of countries compared to the original English version. Region-related differences in how items function have also been found in other utility instruments, such as the EQ-5D [31, 32] and the SF-36 [33]. If utility differences do arise from differences in meaning as a result of translation, then they may very well disappear when using country-specific weights on the respective national data, which would be subject to the same translation effect. In fact, combined evidence of investigations by Scott et al. do suggest an important role of the lack of translation equivalence but also cultural DIF cannot be excluded. Further investigation on these issues is warranted, especially on the relative contribution of translation versus real differences in culture-specific attitudes towards health. Although these are complex topics to research, they are the key to truly understanding the inter-country differences we also have observed in the valuations presented here. A standardised and evaluated QLU-C10D valuation methodology is being used we will in future be able study utility differences between countries in a more sophisticated way.

A potential limitation of our study is that levels of education in the samples were slightly higher than in the respective general populations. The same was observed in the Australian valuation study [9] and is typical for online panels [34]. However, this would only be a problem if health valuation differed by education level. There is evidence that education level does not affect health valuation in different time trade of tasks [35, 36], however, this has not yet been investigated for DCEs. We intend to explore this with our data, but that is beyond the scope of the current paper.

The results from debriefing questions at the end of the valuation surveys indicated that clarity was not an issue in the vast majority of respondents. Task difficulty ratings do not raise severe concerns but require more caution in interpretation. If DCE tasks are too difficult or too easy they might be countering the required trade-off. The literature on the perceived difficulty of making decisions in health DCEs is somewhat scarce. The numbers we found compare well to the results from the QLU-C10D feasibility study [14] and are well within the range of what has been reported and considered acceptable in the literature so far. Mulhern et al. 2016 directly compared a DCE with a time-trade-off (TTO) for the EQ-5D-5L resulting in 57% considering the DCE tasks difficult to answer and 63% considering the TTO tasks difficult to answer [37]. In a study by Norman et al. (2013), a much lower number of 11% of respondents rated tasks of a DCE for the valuation of EQ-5D-5L to be either difficult or very difficult [38]. Other examples of DCEs in the context of health are Skedgel et al. (2013) who investigated societal preferences for the allocation of health care resources an found 65% to rate the presented DCE questions somewhat or extremely difficult to answer [39], and Green and Gerard (2009), likewise a societal DCE, where as many as 68% considered it fairly difficult or very difficult to complete the DCE tasks [40].

We conclude that the QLU-C10D enables the incorporation of QOL data collected via the QLQ-C30 into economic evaluation in Europe. This is especially important when QOL is a significant outcome of an investigated health intervention. Based on our results, we advise the use of country-specific QLU-C10D value sets for the evaluation of treatment effects whenever possible. In line with a recent suggestion recently also made for the EQ-5D [41], we advocate further investigation and discussion of the compatibility of translations and value sets, especially when used in multinational clinical studies. Future research will show how QLU-C10D cancer-specific utility values compare to generic ones in terms of sensitivity and responsiveness to clinical differences, and whether the choice of instrument will impact cost-utility ratios.

## Electronic supplementary material

Below is the link to the electronic supplementary material.

Online Resource 1 Fig. A1: Results of Discrete Choice Experiment (DCE) feedback questions in percentages Supplementary file1 (PDF 37 kb)

Online Resource 2 Table A2: Unadjusted utility model raw scores for Austria, Italy, and Poland Supplementary file2 (DOCX 19 kb)

Supplementary file3 (PDF 120 kb)

Supplementary file4 (PDF 120 kb)

Supplementary file5 (PDF 120 kb)

## References

[1] Dolan P (1996). The time trade-off method: results from a general population study. Health Economics.

[2] Herdman M (2011). Development and preliminary testing of the new five-level version of EQ-5D (EQ-5D-5L). Quality of Life Research.

[3] Brazier J, Roberts J, Deverill M (2002). The estimation of a preference-based measure of health from the SF-36. J Health Econ.

[4] Brazier JE (2012). Developing and testing methods for deriving preference-based measures of health from condition-specific measures (and other patient-based measures of outcome). Health Technology Assessment.

[5] EUnetHTA. EUnetHTA JA1 WP5. Endpoints used for relative effe ctiveness assessment of pharmaceuticals. Health-related quality of life and ut ility measures. 2013 24.06.2019]; Available from: https://www.eunethta.eu/wp-content/uploads/2013/01/Health-related-quality-of-life.pdf.

[6] King MT (2016). QLU-C10D: a health state classification system for a multi-attribute utility measure based on the EORTC QLQ-C30. Quality of Life Research.

[7] Aaronson NK (1993). The European Organization for Research and Treatment of Cancer QLQ-C30: a quality-of-life instrument for use in international clinical trials in oncology. Journal of the National Cancer Institute.

[8] Smith AB (2014). Reporting of health-related quality of life (HRQOL) data in oncology trials: a comparison of the European Organization for Research and Treatment of Cancer Quality of Life (EORTC QLQ-C30) and the Functional Assessment of Cancer Therapy-General (FACT-G). Quality of Life Research.

[9] King MT (2018). Australian Utility Weights for the EORTC QLU-C10D, a Multi-Attribute Utility Instrument Derived from the Cancer-Specific Quality of Life Questionnaire, EORTC QLQ-C30. Pharmacoeconomics.

[10] McTaggart-Cowan H (2019). The EORTC QLU-C10D: The Canadian Valuation Study and Algorithm to Derive Cancer-Specific Utilities From the EORTC QLQ-C30. MDM Policy Pract.

[11] Kemmler, G., et al., German value sets for the EORTC QLU-C10D, a cancer-specific utility instrument based on the EORTC QLQ-C30 Quality of Life Research, (accepted August 2019). 10.1007/s11136-019-02283-w PMC6863792 31485913

[12] Norman, R., et al., U.K. utility weights for the EORTC QLU-C10D. Health Econ, 2019. 10.1002/hec.3950 31482619

[13] Kessler RC (2002). Short screening scales to monitor population prevalences and trends in non-specific psychological distress. Psychological Medicine.

[14] Norman R (2016). Using a discrete choice experiment to value the QLU-C10D: feasibility and sensitivity to presentation format. Quality of Life Research.

[15] Norman R (2016). Order of Presentation of Dimensions Does Not Systematically Bias Utility Weights from a Discrete Choice Experiment. Value Health.

[16] Gamper EM (2018). Test-Retest Reliability of Discrete Choice Experiment for Valuations of QLU-C10D Health States. Value Health.

[17] Aaronson NK (1993). Assessment of quality of life and benefits from adjuvant therapies in breast cancer. Recent Results in Cancer Research.

[18] Scott NW (2013). An evaluation of the response category translations of the EORTC QLQ-C30 questionnaire. Quality of Life Research.

[19] Deutsches Register klinischer Studien. Untersuchung der deutschsprachigen Antwortskala des Fragebogens zur Lebensqualität der European Organisation for Research and Treatment of Cancer (EORTC QLQ-C30) – ein Drei-Stufen Ansatz. 2017 August 21, 2018]; Available from: https://www.drks.de/drks_web/navigate.do?navigationId=trial.HTML&TRIAL_ID=DRKS00012759.

[20] Bevölkerungspyramide Deutschland. Available from: https://service.destatis.de/bevoelkerungspyramide/#

[21] Statistik Austria. (retrieved 23/01/2018)]; Available from: https://www.statistik.at/web_de/statistiken/menschen_und_gesellschaft/bevoelkerung/index.html.

[22] Istat. retrieved 07/2017]; Available from: https://demo.istat.it/pop2017/index_e.html.

[23] Index Mundi. Available from: https://www.indexmundi.com/poland/age_structure.html.

[24] Statistics Poland [cited 2018; Available from: https://stat.gov.pl/en/topics/population/population/structure-of-the-population-by-2016,7,1.html.

[25] Eurostat. [cited 2018; Available from: https://appsso.eurostat.ec.europa.eu/nui/submitViewTableAction.do.

[26] Bansback N (2012). Using a discrete choice experiment to estimate health state utility values. J Health Econ.

[27] Mulhern B (2019). One Method, Many Methodological Choices: A Structured Review of Discrete-Choice Experiments for Health State Valuation. Pharmacoeconomics.

[28] EUnetHTA. EUnetHTA methodological guideline – Methods for health economic evaluations. 2015 24.06.2019]; Available from: https://www.eunethta.eu/wp-content/uploads/2018/01/SAG-_Public_consultation_table-with-comments-and-answers_ECO-GL_final.pdf.

[29] Scott NW (2006). Comparing translations of the EORTC QLQ-C30 using differential item functioning analyses. Quality of Life Research.

[30] Scott NW (2007). The use of differential item functioning analyses to identify cultural differences in responses to the EORTC QLQ-C30. Quality of Life Research.

[31] Whynes DK (2013). Testing for differential item functioning within the EQ-5D. Medical Decision Making.

[32] Salomon JA (2011). Comparability of patient-reported health status: multicountry analysis of EQ-5D responses in patients with type 2 diabetes. Medical Care.

[33] Bath PM (2001). Tinzaparin in acute ischaemic stroke (TAIST): a randomised aspirin-controlled trial. Lancet.

[34] Hays RD, Liu H, Kapteyn A (2015). Use of Internet panels to conduct surveys. Behavior Research Methods.

[35] Yang Z (2017). Logical inconsistencies in time trade-off valuation of EQ-5D-5L health states: Whose fault is it?. PLoS ONE.

[36] Jin X (2016). Is bad living better than good death? Impact of demographic and cultural factors on health state preference. Quality of Life Research.

[37] Mulhern B (2016). Valuing Health Using Time Trade-Off and Discrete Choice Experiment Methods: Does Dimension Order Impact on Health State Values?. Value Health.

[38] Norman R, Cronin P, Viney R (2013). A pilot discrete choice experiment to explore preferences for EQ-5D-5L health states. Appl Health Econ Health Policy.

[39] Skedgel CD, Wailoo AJ, Akehurst RL (2015). Choosing vs allocating: discrete choice experiments and constant-sum paired comparisons for the elicitation of societal preferences. Health Expectations.

[40] Green C, Gerard K (2009). Exploring the social value of health-care interventions: a stated preference discrete choice experiment. Health Economics.

[41] Gerlinger C (2019). Comparing the EQ-5D-5L utility index based on value sets of different countries: impact on the interpretation of clinical study results. BMC Res Notes.

