# Supplementary material for: EORTC QLU-C10D value sets for Austria, Italy, and Poland
Source: Qual Life Res. 2020 May 26;29(9):2485–95. doi: 10.1007/s11136-020-02536-z (PMC7434806; doi:10.1007/s11136-020-02536-z)

EORTC QLU-C10D value sets for Austria, Italy, and Poland, Quality of Life Research,

Gamper EM, King MT, Norman R, Efficace F, Cottone F, Holzner B, Kemmler G;
Corresponding author: Eva-Maria Gamper, Department of Psychiatry, Psychotherapy and Psychosomatics,
University Hospital Psychiatry II, Medical University of Innsbruck, Innsbruck, Austria;
eva-maria.gamper@i-med.ac.at

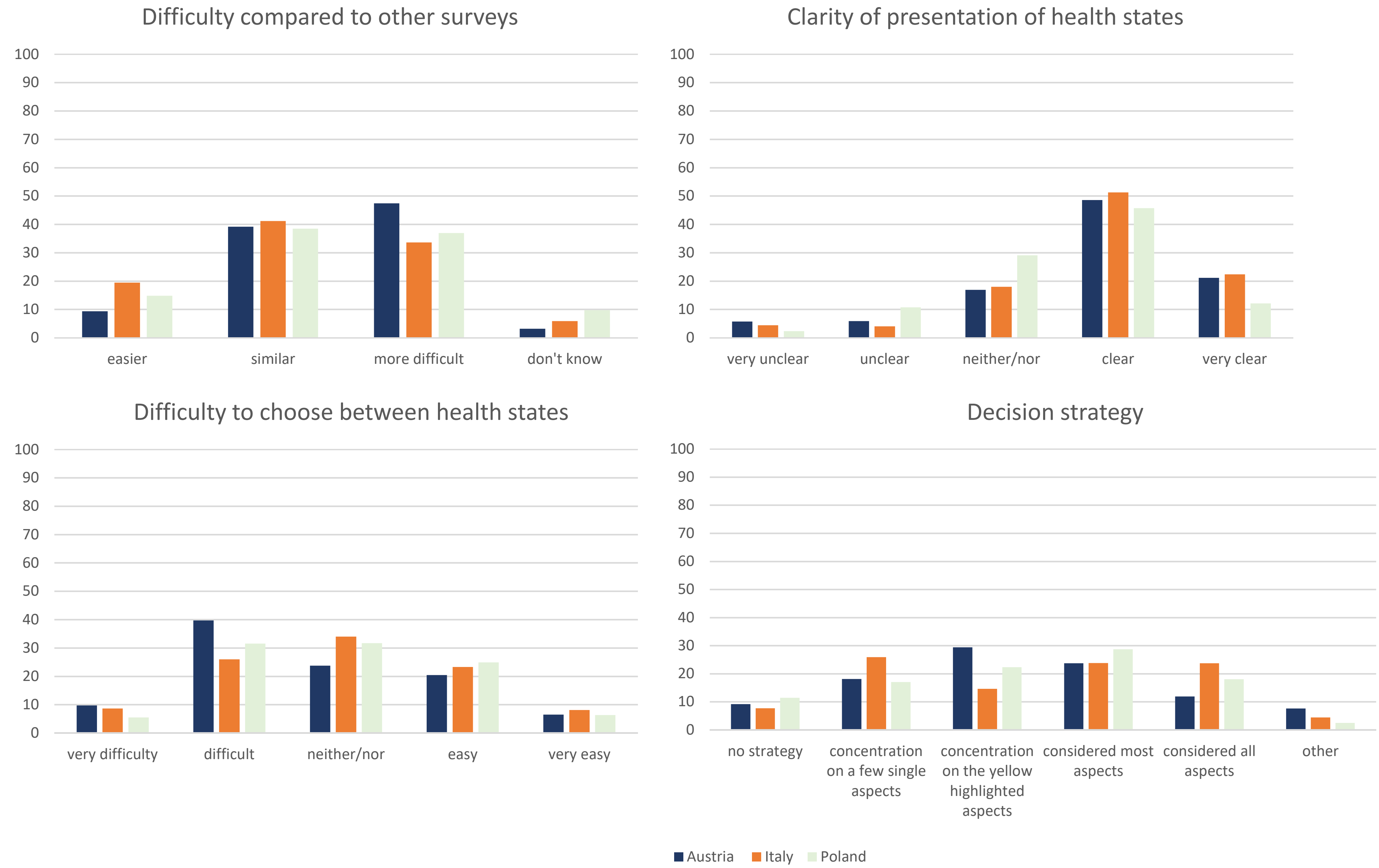

Supplement: Supplementary file 1 — Online Resource 1 Fig. A1: Results of Discrete Choice Experiment (DCE) feedback questions in percentages Supplementary file1 (PDF 37 kb) [file 11136_2020_2536_MOESM1_ESM.pdf]
