# Supplementary material for: EORTC QLU-C10D value sets for Austria, Italy, and Poland
Source: Qual Life Res. 2020 May 26;29(9):2485–95. doi: 10.1007/s11136-020-02536-z (PMC7434806; doi:10.1007/s11136-020-02536-z)
Supplement: Supplementary file 2 — Online Resource 2 Table A2: Unadjusted utility model raw scores for Austria, Italy, and Poland Supplementary file2 (DOCX 19 kb) [file 11136_2020_2536_MOESM2_ESM.docx]

| **Dimension** | **Level** | **Utility decrement^a^ (SE)** | | |
| --- | --- | --- | --- | --- |
|  |  | **Austria^b^** | **Italy** | **Poland** |
| Physical Functioning | 1 (not at all) | 0 | 0 | 0 |
|  | 2 (a little) | -0.117 (0.021) | -0.047 (0.027) | -0.065 (0.025) |
|  | 3 (quite a bit) | -0.233 (0.022) | -0.203 (0.023) | -0.152 (0.026) |
|  | 4 (very much) | -0.316 (0.022) | -0.298 (0.022) | -0.276 (0.025) |
|  |  |  |  |  |
| Role Functioning | 1 (not at all) | 0 | 0 | 0 |
|  | 2 (a little) | -0.012 (0.017) | -0.017 (0.018) | -0.071 (0.020) |
|  | 3 (quite a bit) | -0.076 (0.016) | -0.076 (0.018) | -0.140 (0.023) |
|  | 4 (very much) | -0.139 (0.015) | -0.117 (0.017) | -0.197 (0.021) |
|  |  |  |  |  |
| Social Functioning | 1 (not at all) | 0 | 0 | 0 |
|  | 2 (a little) | -0.001 (0.017) | -0.004 (0.018) | 0.020 (0.020) |
|  | 3 (quite a bit) | -0.073 (0.016) | -0.042 (0.018) | 0.003 (0.023) |
|  | 4 (very much) | -0.103 (0.015) | -0.044 (0.016) | -0.025 (0.019) |
|  |  |  |  |  |
| Emotional Functioning | 1 (not at all) | 0 | 0 | 0 |
|  | 2 (a little) | -0.003 (0.015) | 0.001 (0.019) | -0.003 (0.020) |
|  | 3 (quite a bit) | 0.005 (0.017) | -0.068 (0.019) | -0.019 (0.021) |
|  | 4 (very much) | -0.036 (0.013) | -0.118 (0.016) | -0.034 (0.018) |
|  |  |  |  |  |
| Pain | 1 (not at all) | 0 | 0 | 0 |
|  | 2 (a little) | -0.036 (0.016) | -0.014 (0.018) | -0.015 (0.020) |
|  | 3 (quite a bit) | -0.112 (0.016) | -0.077 (0.018) | -0.069 (0.021) |
|  | 4 (very much) | -0.183 (0.016) | -0.128 (0.017) | -0.127 (0.018) |
|  |  |  |  |  |
| Fatigue | 1 (not at all) | 0 | 0 | 0 |
|  | 2 (a little) | -0.028 (0.015) | -0.013 (0.017) | -0.012 (0.020) |
|  | 3 (quite a bit) | -0.048 (0.015) | -0.061 (0.019) | -0.044 (0.020) |
|  | 4 (very much) | -0.057 (0.014) | -0.061 (0.016) | -0.041 (0.018) |
|  |  |  |  |  |
| Sleep disturbances | 1 (not at all) | 0 | 0 | 0 |
|  | 2 (a little) | -0.022 (0.014) | -0.030 (0.016) | -0.021 (0.019) |
|  | 3 (quite a bit) | -0.034 (0.016) | -0.057 (0.018) | -0.024 (0.020) |
|  | 4 (very much) | -0.039 (0.014) | -0.040 (0.016) | -0.037 (0.018) |
|  |  |  |  |  |
| Appetite loss | 1 (not at all) | 0 | 0 | 0 |
|  | 2 (a little) | -0.048 (0.014) | -0.024 (0.016) | -0.015 (0.019) |
|  | 3 (quite a bit) | -0.048 (0.015) | -0.027 (0.018) | -0.049 (0.020) |
|  | 4 (very much) | -0.061 (0.013) | -0.019 (0.016) | -0.053 (0.019) |
|  |  |  |  |  |
| Nausea | 1 (not at all) | 0 | 0 | 0 |
|  | 2 (a little) | -0.028 (0.014) | -0.036 (0.016) | -0.037 (0.018 |
|  | 3 (quite a bit) | -0.073 (0.016) | -0.081 (0.017) | -0.056 (0.020 |
|  | 4 (very much) | -0.108 (0.014) | -0.088 (0.016) | -0.084 (0.018 |
|  |  |  |  |  |
| Bowel Problems | 1 (not at all) | 0 | 0 | 0 |
|  | 2 (a little) | -0.022 (0.015) | -0.023 (0.017) | -0.034 (0.019) |
|  | 3 (quite a bit) | -0.061 (0.015) | -0.028 (0.017) | -0.067 (0.020) |
|  | 4 (very much) | -0.069 (0.014) | -0.051 (0.016) | -0.076 (0.018) |

EORTC QLU-C10D value sets for Austria, Italy, and Poland, Quality of Life Research,

Gamper EM, King MT, Norman R, Efficace F, Cottone F, Holzner B, Kemmler G;

Corresponding author: Eva-Maria Gamper, Department of Psychiatry, Psychotherapy and Psychosomatics,

University Hospital Psychiatry II, Medical University of Innsbruck, Innsbruck, Austria;

eva-maria.gamper@i-med.ac.at

Table A2: Unadjusted utility model raw scores for Austria, Italy, and Poland
