# Supplementary material for: EORTC QLU-C10D value sets for Austria, Italy, and Poland
Source: Qual Life Res. 2020 May 26;29(9):2485–95. doi: 10.1007/s11136-020-02536-z (PMC7434806; doi:10.1007/s11136-020-02536-z)
Supplement: Supplementary file 3 — Supplementary file3 (PDF 120 kb) [file 11136_2020_2536_MOESM3_ESM.pdf]

EORTC QLU-C10D value sets for Austria, Italy, and Poland, Quality of Life Research,
Gamper EM, King MT, Norman R, Efficace F, Cottone F, Holzner B, Kemmler G;
Corresponding author: Eva-Maria Gamper, Department of Psychiatry, Psychotherapy and Psychosomatics,
University Hospital Psychiatry II, Medical University of Innsbruck, Innsbruck, Austria;
eva-maria.gamper@i-med.ac.at

```
********************************************************************************
****************************************
* Example code for converting EORTC QLQ-C30 data into QLU-C10D utility scores
                                            *
* written for SPSS by Eva Gamper, February 2020
                                           *
*
*
* For further details of the QLU-C10D, see the following papers:
                                            *
*
* King MT, et al Derivation of the health state classification system for the
                                            *
* QLU-C10D, an internationally-valid cancer-specific multi-attribute utility
                                            *
* instrument derived from the EORTC core quality of life questionnaire, QLQ-C30.
                                            *
* Quality of Life Research. 2016; 25(3): 625-636. DOI 10.1007/s11136-015-1217-y
                                            *
*
* Norman R, et al. Using a discrete choice experiment to value the QLU-C10D:
                                            *
* Feasibility and sensitivity to presentation format. Quality of Life Research.
                                            *
* 2016; 25(3): 637-649. DOI 10.1007/s11136-015-1115-3
                                            *
*
* The utility algorithms reported in this code are based on the monotonitcity
adjusted                                    *
* values as reported by Gamper et al. in "EORTC QLU-C10D value sets for Austria,
Italy, and Poland"                          *
*
* This code is written for SPSS users, and notes are added throughout to allow
                                    *
* conversion to other software as required.
                                           *
********************************************************************************
*****************************************

********************************************************************************
***************************
* Assumption: For this codes to work, it is assumed that the EORTC QLQ-C30 code
                                            *
* is set up as thirty columns, labelled qlq1-qlq30 (in the order as given in the
```

```
questionnaire),
*each of which can take one  of four values 1-4, where 1 = "Not at all", 2 = "A little",                                          *
* 3 = "Quite a  bit" and 4 = "Very much". To derive the QLU-C10D, we only need 13 of these                                       *
* Seven of QLU_C10D items are single items from the EORTC QLQ-C30, and three                                          *
* (pf, sf, bo) are composite which are combined                                          *
*******************************************************************************************************************


***************************************************************************************************************************
*QLU-C10D scoring algorithm for AUSTRIA V2 (CAVE! version with response category 3="ziemlich")                          *
***************************************************************************************************************************

IF (qlq2=1) pf = 0 .
IF (qlq2>1) pf = 0.117 .
IF (qlq3>1) pf = 0.234 .
IF (qlq3>2) pf = 0.316 .
EXECUTE.

IF (qlq6=1) rf = 0 .
IF (qlq6=2) rf = 0.012  .
IF (qlq6=3) rf = 0.075 .
IF (qlq6=4) rf = 0.138 .
EXECUTE.

IF (qlq26=1 & qlq27=1) sf = 0 .
IF (qlq26=2 | qlq27=2) sf  = 0.00 .
IF (qlq26=3 | qlq27=3) sf  = 0.072 .
IF (qlq26=4 | qlq27=4) sf  = 0.103 .
EXECUTE.

IF (qlq24=1) ef = 0 .
IF (qlq24=2) ef = 0 .
IF (qlq24=3) ef = 0.  .
IF (qlq24=4) ef = 0.038 .
EXECUTE.

IF (qlq9=1) pa = 0 .
IF (qlq9=2) pa = 0.036  .
IF (qlq9=3) pa = 0.112 .
IF (qlq9=4) pa = 0.182 .
EXECUTE.

IF (qlq18=1) fa = 0 .
IF (qlq18=2) fa = 0.028 .
IF (qlq18=3) fa = 0.048 .
IF (qlq18=4) fa = 0.057 .
```

```
EXECUTE.

IF (qlq11=1) sl = 0 .
IF (qlq11=2) sl = 0.022 .
IF (qlq11=3) sl = 0.034 .
IF (qlq11=4) sl = 0.039 .
EXECUTE.

IF (qlq13=1) ap = 0 .
IF (qlq13=2) ap = 0.049 .
IF (qlq13=3) ap = 0.049 .
IF (qlq13=4) ap = 0.061 .
EXECUTE.

IF (qlq14=1) na = 0 .
IF (qlq14=2) na = 0.029 .
IF (qlq14=3) na = 0.074 .
IF (qlq14=4) na = 0.108 .
EXECUTE.

IF (qlq16=1 & qlq17=1) bo = 0 .
IF (qlq16=2 | qlq17=2) bo = 0.022 .
IF (qlq16=3 | qlq17=3) bo = 0.061 .
IF (qlq16=4 | qlq17=4) bo = 0.069 .
EXECUTE.


COMPUTE QLUC10D_AUTV2 = 1- (pf + rf + sf + ef + pa + fa + sl + ap + na + bo) .
FORMATS QLUC10D_AUTV2 (F8.3).
EXECUTE.

********************************************************************************
* The new variable QLUC10D_AUTV2 is a utility score where full health
(i.e. level 1  in each of the utility levels) is scored at 1, and the minimum
score
(i.e. each utility level is at 4) is -0.111. These data can now be used to
* construct quality-adjusted life years (QALYs) for cost-utility analysis.
********************************************************************************
```
